# Do Judge a Test by its Cover: Combining Combinatorial and Property-Based Testing

**DOI:** 10.1007/978-3-030-72019-3_10

**Published:** 2021-03-23

**Authors:** Harrison Goldstein, John Hughes, Leonidas Lampropoulos, Benjamin C. Pierce

**Affiliations:** Imperial College, London, UK; 9 University of Pennsylvania, Philadelphia, PA 19104 USA; 10 Chalmers University of Technology and Quviq AB, 412 96 Gothenburg, Sweden; 11 University of Maryland, College Park, MD 20742 USA

**Keywords:** Combinatorial testing, Combinatorial coverage, QuickCheck, Property-based testing, Regular tree expressions, Algebraic data types

## Abstract

*Property-based testing* uses randomly generated inputs to validate high-level program specifications. It can be shockingly effective at finding bugs, but it often requires generating a very large number of inputs to do so. In this paper, we apply ideas from *combinatorial testing*, a powerful and widely studied testing methodology, to modify the distributions of our random generators so as to find bugs with fewer tests. The key concept is *combinatorial coverage*, which measures the degree to which a given set of tests exercises every possible choice of values for every small combination of input features.

In its “classical” form, combinatorial coverage only applies to programs whose inputs have a very particular shape—essentially, a Cartesian product of finite sets. We generalize combinatorial coverage to the richer world of algebraic data types by formalizing a class of *sparse test descriptions* based on regular tree expressions. This new definition of coverage inspires a novel *combinatorial thinning* algorithm for improving the coverage of random test generators, requiring many fewer tests to catch bugs. We evaluate this algorithm on two case studies, a typed evaluator for System F terms and a Haskell compiler, showing significant improvements in both.

### 1 Introduction

Property-based testing, popularized by tools like *QuickCheck* [7], is a principled way of testing software that focuses on functional specifications rather than suites of input-output examples. A property is a formula like

$$\forall x.\ P(x,\ f(x)),$$

* For the full version, including all appendices, visit `https://harrisongoldste.in/papers/quick-cover.pdf`.

✉ `hgo@seas.upenn.edu`

where $f$ is the function under test and $P$ is some executable logical relationship between an input $x$ and the output $f(x)$. The test harness generates random values for $x$, hoping to either uncover a counterexample—an $x$ for which $\neg P(x,\ f(x)))$, indicating a bug—or else provide confidence that $f$ is correct with respect to $P$.

With a well-designed random test case generator, property-based testing has a non-zero probability of generating *every* valid test case (up to a given size limit); property-based testing is thus guaranteed to find any bug that can be provoked by an input below the size limit... eventually. Unfortunately, since each input is generated independently, random testing may end up repeating the same or similar tests many times before happening across the specific input which provokes a bug. This poses a particular problem in settings like *continuous integration*, where feedback is needed quickly—it would be nice to have an automatic way to guide the generator to a more interesting and diverse set of inputs, "thinning" the distribution to find bugs with fewer tests.

Combinatorial testing, an elegant approach to testing from the software engineering literature [2,16,17], offers an attractive metric for judging which tests are most interesting. In its classical presentation, combinatorial testing advocates choosing tests to maximize *t-way coverage* of a program's input space—i.e., to exercise all possible choices of concrete values for every combination of $t$ input parameters. For example, suppose a program $p$ takes Boolean parameters `w`, `x`, `y`, and `z`, and suppose we want to test that $p$ behaves well for every choice of values for every pair of these four parameters. If we choose carefully, we can check all such choices—all 2-*way interactions*—with just five test cases:

| | | | | |
|---|---|---|---|---|
| 1. | `w = False` | `x = False` | `y = False` | `z = False` |
| 2. | `w = False` | `x = True` | `y = True` | `z = True` |
| 3. | `w = True` | `x = False` | `y = True` | `z = True` |
| 4. | `w = True` | `x = True` | `y = False` | `z = True` |
| 5. | `w = True` | `x = True` | `y = True` | `z = False` |

You can check for yourself: for any two parameters, every combination of values for these parameters is covered by some test. For example, "`w = False` and `x = False`" is covered by #1, while both "`w = True` and `x = True`" and "`w = True` and `y = True`" are covered by #5. Any other test case we could come up with would check a redundant set of 2-way interactions. Thus, we get 100% pairwise coverage with just five out of the $2^4 = 16$ possible inputs. This advantage improves exponentially with the number of parameters.

Why is this interesting? Because surveys of real-world systems have shown that bugs are often provoked by specific choices of just a few parameters [16]. Indeed, one study involving a distributed database at NASA found that, out of 100 known failures, 93 were caused by 2-way parameter interactions; the remaining 7 failures were each caused by no more than 6 parameters interacting together [14]. This suggests that combinatorial testing is an effective way to choose test cases for real systems.

If combinatorial coverage can be used to concentrate bug-finding power into small sets of tests, it is natural to wonder whether it could also be used to thin the distribution of a random generator. So far, combinatorial testing has mostly been applied in settings where the input to a program is just a vector of parameters, each drawn from a small finite set. Could we take it further? In particular, could we transfer ideas from combinatorial testing to the richer setting addressed by *QuickCheck*—i.e., functional programs whose inputs are drawn from structured, potentially infinite data types like lists and trees?

Our first contribution is showing how to generalize the definition of combinatorial coverage to work with *regular tree expressions*, which themselves generalize the algebraic data types found in most functional languages. Instead of covering combinations of parameter choices, we measure coverage of *test descriptions*—concise representations of sets of tests, encoding potentially interesting interactions between data constructors. For example, the test description cons(true, ◇false) describes the set of Boolean lists that have true as their first element, followed by at least one false somewhere in the tail.

Our second contribution is a method for enhancing property-based testing using combinatorial coverage. We propose an algorithm that uses combinatorial coverage information to thin an existing random generator, leading it to more interesting test suites that find bugs more often. A concrete realization of this algorithm in a tool called *QuickCover* was able, in our experiments, to guide random generation to find bugs using an average of 10× fewer tests than *QuickCheck*. While *generating* test suites is (considerably) slower, *running* the tests can be much faster. As such, *QuickCover* excels in settings where tests are particularly costly to run, as well as in situations like continuous-integration, when the cost of test generation is amortized over many runs of the test suite.

In summary, we offer these contributions:

- We generalize the notion of combinatorial coverage to work over a set of *test descriptions* and show how this new definition generalizes to algebraic data types with the help of regular tree expressions (Section 3). Section 4 describes the technical details behind the specific way we choose to represent these descriptions.
- We propose a process for guiding the test distribution of an existing random generator based on our generalized notion of combinatorial coverage (Section 5).
- Finally, we demonstrate, with two case studies, that *QuickCover* can find bugs using significantly fewer tests (Section 6) than pure random testing.

We conclude with an overview of related work (Section 7), and ideas for future work (Section 8).

### 2 Classical Combinatorial Testing

To set the stage, we begin with a brief review of "classical" combinatorial testing.

Combinatorial testing measures the "combinatorial coverage" of test suites, aiming to find more bugs with fewer tests. Standard presentations [16] are phrased in terms of a number of separate input parameters. Here, for notational consistency with the rest of the paper, we will instead assume that a program takes a single input consisting of a tuple of values.

Assume we are given some finite set $\mathcal{C}$ of *constructors*, and consider the set of $n$-tuples over $\mathcal{C}$:

$$\{\mathsf{tuple}_n(C_1, \ldots, C_n) \mid C_1, \ldots, C_n \in \mathcal{C}\}$$

(The “constructor” $\mathsf{tuple}_k$ is not strictly needed in this section, but it makes the generalization to constructor trees and tree regular expressions in Section 3 smoother.) We can use these tuples to represent test inputs to systems. For example a web application might be tested under configurations

$$\mathsf{tuple}_4(\mathsf{Safari},\ \mathsf{MySQL},\ \mathsf{Admin},\ \mathsf{English})$$

in order to verify some end-to-end property of the system.

A *specification* of a set of tuples is written informally using notation like:

$$\mathsf{tuple}_4(\mathsf{Safari}{+}\mathsf{Chrome},\ \mathsf{Postgres}{+}\mathsf{MySQL},\ \mathsf{Admin}{+}\mathsf{User},\ \mathsf{French}{+}\mathsf{English})$$

This specification restricts the set of valid tests to those that have valid browsers in the first position, valid databases in the second, and so on. Specifications are thus a lot like types—they pick out a set of valid tests from some larger set. We define this notation precisely in Section 3.

To define combinatorial coverage, we introduce the notion of *partial* tuples—i.e., tuples where some elements are left indeterminate (written $\top$). For example:

$$\mathsf{tuple}_4(\mathsf{Chrome},\ \top,\ \mathsf{Admin},\ \top).$$

A description is *compatible* with a specification if its concrete (non-$\top$) constructors are valid in the positions where they appear. Thus, the description above is compatible with our web-app configuration specification, while this one is not:

$$\mathsf{tuple}_4(\mathsf{MySQL},\ \mathsf{MySQL},\ \mathsf{French},\ \top)$$

We say a test *covers* a description—which, conversely, *describes* the test—when the tuple matches the description in every position that does not contain $\top$. For example, the description

$$\mathsf{tuple}_4(\mathsf{Chrome},\ \top,\ \mathsf{Admin},\ \top)$$

describes these tests:

$$\mathsf{tuple}_4(\mathsf{Chrome},\ \mathsf{MySQL},\ \mathsf{Admin},\ \mathsf{English})$$
$$\mathsf{tuple}_4(\mathsf{Chrome},\ \mathsf{MySQL},\ \mathsf{Admin},\ \mathsf{French})$$
$$\mathsf{tuple}_4(\mathsf{Chrome},\ \mathsf{Postgres},\ \mathsf{Admin},\ \mathsf{English})$$
$$\mathsf{tuple}_4(\mathsf{Chrome},\ \mathsf{Postgres},\ \mathsf{Admin},\ \mathsf{French})$$

Finally, we call a description *$t$-way* if it fixes exactly $t$ constructors, leaving the rest as $\top$.

Now, suppose a system under test takes a tuple of configuration values as input. Given some correctness property (e.g., the system does not crash), a test for the system is simply a particular tuple, while a test *suite* is a set of tuples. We can then define *combinatorial coverage* as follows:

**Definition 1.** *The $t$-way* combinatorial coverage *of a test suite is the proportion of $t$-way descriptions, compatible with a given specification, that are covered by some test in the suite.*

We say that $t$ is the *strength* of the coverage.

A test suite with 100% 2-way coverage for the present example can be quite small. For example,

$$\begin{array}{l}\mathsf{tuple}_4(\mathsf{Chrome},\ \mathsf{Postgres},\ \mathsf{Admin},\ \mathsf{English})\\ \mathsf{tuple}_4(\mathsf{Chrome},\ \mathsf{MySQL},\ \mathsf{User},\ \mathsf{French})\\ \mathsf{tuple}_4(\mathsf{Safari},\ \mathsf{Postgres},\ \mathsf{User},\ \mathsf{French})\\ \mathsf{tuple}_4(\mathsf{Safari},\ \mathsf{MySQL},\ \mathsf{Admin},\ \mathsf{French})\\ \mathsf{tuple}_4(\mathsf{Safari},\ \mathsf{MySQL},\ \mathsf{User},\ \mathsf{English})\end{array}$$

achieves 100% coverage with just five tests. The fact that a single test covers many different descriptions is what makes combinatorial testing work: while the number of descriptions that must be covered is combinatorially large, a single test can cover combinatorially many descriptions. In general, for a tuple of size $n$, the number of descriptions is given by $\binom{n}{t}$ ways to choose $t$ parameters multiplied by the number of distinct values each parameter can take on.

### 3 Generalizing Coverage

Of course, inputs to programs are often more complex than just tuples of enumerated values, especially in the world of functional programming. To apply the ideas of combinatorial coverage in this richer world, we generalize tuples to *constructor trees* and tuple specifications to *regular tree expressions*. We can then give a generalized definition of test descriptions that makes sense for algebraic data types, setting up for a more powerful definition of combinatorial coverage.

A *ranked alphabet* $\Sigma$ is a finite set of atomic *data constructors*, each with a specified *arity*. For example, the ranked alphabet

$$\Sigma_{\mathsf{list(bool)}} \triangleq \{(\mathsf{cons},\ 2),\ (\mathsf{nil},\ 0),\ (\mathsf{true},\ 0),\ (\mathsf{false},\ 0)\}$$

defines the constructors needed to represent lists of Booleans. Given a ranked alphabet $\Sigma$, the set of *trees* over $\Sigma$ is the least set $\mathcal{T}_\Sigma$ that satisfies the equation

$$\mathcal{T}_\Sigma = \{C(t_1,\ \ldots,\ t_n) \mid (C,\ n) \in \Sigma \wedge t_1,\ \ldots,\ t_n \in \mathcal{T}_\Sigma\}.$$

*Regular tree expressions* are a compact and powerful tool for specifying sets of trees [9,10]. They are generated by the following syntax:

$$
\begin{aligned}
e \triangleq\ & \top \\
& \mid e_1 + e_2 \\
& \mid \mu X.\ e \\
& \mid X \\
& \mid C(e_1,\ \dots,\ e_n) \text{ for } (C,\ n) \in \Sigma
\end{aligned}
$$

Each of these operations has an analog in standard regular expressions over strings: + corresponds to disjunction of regular expressions, $\mu$ corresponds to iteration, and the parent-child relationship corresponds to concatenation. These expressions give us a rich language for describing tree structures.

The *denotation* function $[\![\cdot]\!]$ mapping regular tree expressions to sets of trees is the least function satisfying the equations:

$$
\begin{aligned}
[\![\top]\!] &= \mathcal{T}_\Sigma \\
[\![C(e_1,\ \dots,\ e_n)]\!] &= \{C(t_1,\ \dots,\ t_n) \mid t_i \in [\![e_i]\!]\} \\
[\![e_1 + e_2]\!] &= [\![e_1]\!] \cup [\![e_2]\!] \\
[\![\mu X.\ e]\!] &= [\![e[\mu X.\ e/X]]\!]
\end{aligned}
$$

Regular tree expressions subsume standard first-order algebraic data type definitions. For example, the Haskell definition

```
data BoolList = Cons Bool BoolList | Nil
```

is equivalent to the regular tree expression

$$\mu X.\ \mathsf{cons}(\mathsf{true} + \mathsf{false},\ X) + \mathsf{nil}.$$

Crucially for our purposes, regular tree expressions can also be used to define sets of trees that cannot be described with plain ADTs. For example, the expression

$$\mathsf{cons}(\mathsf{true} + \mathsf{false},\ \mathsf{nil})$$

denotes all single-element Boolean lists, while

$$\mu X.\ \mathsf{cons}(\mathsf{true},\ X) + \mathsf{nil}$$

describes the set of lists that only contain true. Regular tree expressions can even express constraints like "true appears at some point in the list":

$$\mu X.\ \mathsf{cons}(\top,\ X) + \mathsf{cons}(\mathsf{true},\ \mu Y.\ \mathsf{cons}(\top,\ Y) + \mathsf{nil})$$

This machinery smoothly generalizes the structures we saw in Section 3. Tuples are just a special form of trees, while specifications and test descriptions can be written as regular tree expressions. This gives us most of what we need to define algebraic data types.

Recall the definition of $t$-way combinatorial coverage: "the proportion of (1) $t$-way descriptions, (2) compatible with a given specification, that (3) are covered by some test in the suite." What does this mean in the context of regular tree expressions and trees?

Condition (3) is easy: a test (i.e., a tree) $t$ covers a test description (a regular tree expression) $d$ if $t \in [\![d]\!]$.

For (2), consider some regular tree expression $\tau$ representing an algebraic data type that we would like to cover. We say that a description $d$ is compatible with $\tau$ if $[\![\tau]\!] \cap [\![d]\!] \neq \varnothing$. As with string regular expressions, this can be checked efficiently.

The only remaining question is (1): which set of $t$-way descriptions to use. We argue in the next section that the set of *all* regular tree expressions is too broad, and we offer a simple and natural alternative.

### 4 Sparse Test Descriptions

A naïve way to generalize the definition of $t$-way descriptions to regular tree expressions would be to first define the *size* of a regular tree expression as the number of operators (constructors, $+$, or $\mu$) in it and then define a $t$-way description to be any regular tree expression of size $t$. However, this approach does not specialize nicely to the classical case; for example the description

$$\mathsf{tuple}_4(\mathsf{Safari} + \mathsf{Chrome},\ \top,\ \top,\ \top)$$

would be counted as "4-way" (3 constructors and 1 "+" operator), even though it is covered by *every* well-formed test. Worse, "interesting" descriptions are often quite large. For example, the smallest possible description of lists in which true is followed by false,

$$\mu X.\ \mathsf{cons}(\top,\ X) + \mathsf{cons}(\mathsf{true},\ \mu Y.\ \mathsf{cons}(\top,\ Y) + \mathsf{cons}(\mathsf{false},\ \mu Z.\ \mathsf{cons}(\top,\ Z) + \mathsf{nil}))$$

has size $t = 14$. We want a representation that packs as much information as possible into small descriptions, making $t$-way coverage meaningful for small values of $t$ and increasing the complexity of the interactions captured by our definition of coverage.

In sum, we want a definition of coverage that straightforwardly specializes to the tuples-of-constructors case and that captures interesting structure with small descriptions.

Our proposed solution, described next, takes inspiration from temporal logic. We first encode an "eventually" ($\diamond$) operator that allows us to write the expression from above much more compactly as $\diamond\mathsf{cons}(\mathsf{true},\ \diamond\mathsf{false})$. This can be read as "somewhere in the tree, there is a cons node with a true node to its left and a false node somewhere in the tree to its right." Then we define a restricted form of *sparse test descriptions* using just $\diamond$, $\top$, and constructors.

#### 4.1 Encoding "Eventually"

The "eventually" operator can actually be encoded using the regular tree expression operators we have already defined—i.e., we can add it without adding any formal power. First, define the set of *templates* for the ranked alphabet $\Sigma$:

$$\mathbb{T} \triangleq \{C(\top_1,\ \ldots,\ \top_{i-1},\ [],\ \top_{i+1}\ldots,\ \top_n) \mid (C,\ n) \in \Sigma,\ 1 \leq i \leq n\}$$

That is, for each constructor $C$ in $\Sigma$, the set of templates $\mathbb{T}$ contains $C([],\ \top,\ \ldots,\ \top)$, $C(\top,\ [],\ \top,\ \ldots,\ \top)$, etc., all the way to $C(\top,\ \ldots,\ \top,\ [])$, enumerating every way to place one hole in the constructor and fill every other argument slot with $\top$. (Nullary constructors are ignored.) Then we define "next" ($\circ e$) and "eventually" ($\diamond e$) as

$$\circ e \triangleq \sum_{T \in \mathbb{T}} T[e]$$

$$\diamond e \triangleq \mu X.\ e + \circ X$$

where $T[e]$ is the replacement of $[\,]$ in $T$ with $e$.[3] Intuitively, $\circ e$ describes any tree $C(t_1,\ \ldots,\ t_n)$ in which $e$ describes some direct child (i.e., $t_1$, $t_2$, and so on), while $\diamond e$ describes anything described by $e$, plus (unrolling the $\mu$) anything described by $\circ e$, $\circ\circ e$, and so on.

This is not the only way to design a compact, expressive subset of regular tree expressions, but our evaluation shows that this has useful properties. In addition, the $\diamond$ notation gives an elegant way to write descriptions like the one from the previous section ($\diamond$cons(true, $\diamond$false),), neatly capturing "somewhere in the tree" constraints that would require many more symbols in the bare language of regular tree expressions.

#### 4.2 Defining Coverage

Even in the language with just $\diamond$, $\top$, and constructors, there is still a fair amount of freedom in how we define the set of $t$-way descriptions. In this section we present one possibility that we have found to be useful in practice; in Section 8 we discuss another interesting option.

The set of *sparse test descriptions* for a given $\Sigma$ is the trees generated by

$$\begin{aligned} d \triangleq\ & \top \\ & \mid \diamond C(d_1,\ \ldots,\ d_n) \text{ for } (C,\ n) \in \Sigma, \end{aligned}$$

that is, trees consisting of constructors prefixed by $\diamond$ and $\top$. We call these descriptions "sparse" because they match specific ancestor-descendant arrangements of

[3] This construction is why we choose to deal with finite ranked alphabets: if $\Sigma$ were infinite, $\mathbb{T}$ would be infinite, and $\circ e$ would be an infinite term that is not expressible as a standard regular tree expression.

constructors but place no restriction on the constructors in between, due to the "eventually" before each constructor.

Sparse test descriptions are designed to be compact, useful in practice, and compatible with the classical definition of coverage. For that reason we aim to keep them as information-dense as possible. First, we do not include the $\mu$ operator directly, instead relying on $\diamond$: indeed, $\diamond$ captures a pattern of recursion that is general enough to express interesting non-local constraints while keeping description complexity low. Similarly, we do not need to include the + operator: any test that covers any test that covers either $C(d_1, \ldots, d_n)$ or $D(d_1, \ldots, d_m)$ will also necessarily cover $C(d_1, \ldots, d_n) + D(d_1, \ldots, d_m)$.

Removing explicit uses of $\mu$ and + does limit the expressive power of sparse test descriptions a little—for example it rules out complex mutually recursive definitions. However, we do not intend to use descriptions to specify entire languages, only fragments of languages that we hope to cover with testing. Naturally, there are many other possible formats for test descriptions that would be interesting to explore—we leave that for future work. In this paper, we chose to make descriptions very compact while preserving most of their expressive power, and the case studies in Section 6 demonstrate that such a choice works well in at least two challenging domains that are relevant to programming languages as a whole.

Finally, we define the size of a description based on the number of constructors it contains. Intuitively, a $t$-way description is one with $t$ constructors; however, in order to be consistent with the classical definition, we omit constructors whose types permit no alternatives. For example, all of the tuple constructors (e.g. $\mathsf{tuple}_4$ in our running example) are left out of the size calculation. This makes $t$-way sparse test description coverage specialize to exactly classical $t$-way parameter interaction coverage for the case of tuples of sums of nullary constructors.

Sparse descriptions work as expected for types like

$$\mathsf{tuple}_4(\mathsf{Safari}{+}\mathsf{Chrome},\ \mathsf{Postgres}{+}\mathsf{MySQL},\ \mathsf{Admin}{+}\mathsf{User},\ \mathsf{French}{+}\mathsf{English}).$$

Despite some stray occurrences of $\diamond$, as in

$$\diamond\mathsf{tuple}_4(\diamond\mathsf{Chrome},\ \diamond\mathsf{MySQL},\ \top,\ \top),$$

the descriptions still describe the same sets of tests as the standard tuple descriptions without the uses of $\diamond$. Thus, our new definition of combinatorial coverage generalizes the classical one.

These descriptions capture a rich set of test constraints in a compact form. The real proof of this is in our evaluation results—see Section 6 for those—but a few more examples may help illustrate.

*Boolean Lists* As a first example, consider the type of Boolean lists:

$$\tau_{\mathsf{list(bool)}} \triangleq \mu X.\ \mathsf{cons}(\mathsf{true} + \mathsf{false},\ X) + \mathsf{nil}.$$

The set of all 2-way descriptions that are compatible with $\tau_{\mathsf{list(bool)}}$ is:

$$\begin{array}{ccc} \diamond\mathsf{cons}(\diamond\mathsf{true},\ \top) & \diamond\mathsf{cons}(\diamond\mathsf{false},\ \top) & \diamond\mathsf{cons}(\top,\ \diamond\mathsf{nil}) \\ \diamond\mathsf{cons}(\top,\ \diamond\mathsf{cons}(\top,\ \top)) & \diamond\mathsf{cons}(\top,\ \diamond\mathsf{true}) & \diamond\mathsf{cons}(\top,\ \diamond\mathsf{false}) \end{array}$$

Unpacking the notation, $\diamond\mathsf{cons}(\diamond\mathsf{true},\ \top)$ describes the set of trees where "at some point in the tree there is a $\mathsf{cons}$ node with a $\mathsf{true}$ node somewhere in its left child."

*Arithmetic Expressions* Consider the type of simple arithmetic expressions over the constants $\mathsf{0}$, $\mathsf{1}$, and $\mathsf{2}$:

$$\tau_{\mathsf{expr}} \triangleq \mu X.\ \mathsf{add}(X,\ X) + \mathsf{mul}(X,\ X) + \mathsf{0} + \mathsf{1} + \mathsf{2}.$$

This type has 2-way descriptions like

$$\diamond\mathsf{add}(\diamond\mathsf{mul}(\top,\ \top),\ \top) \text{ and } \diamond\mathsf{mul}(\top,\ \diamond\mathsf{add}(\top,\ \top)),$$

which capture different nestings of addition and multiplication.

*System F* For a more involved example, let's look at some 2-way sparse descriptions for a much more complex data structure: terms of the polymorphic lambda calculus, System F.

$$\begin{aligned} \tau &\triangleq \mathcal{U} \mid \tau_1 \to \tau_2 \mid n \mid \forall.\tau \\ e &\triangleq () \mid n \mid \lambda\tau.\ e \mid (e_1\ e_2) \mid \Lambda.\, e \mid (e\ \tau) \end{aligned}$$

(We use de Bruijn indices for variable binding, meaning that each variable occurrence in the syntax tree is represented by a natural number indicating which enclosing abstraction it was bound by.)

System F syntax can be represented using a regular tree expression like

$$\mu X.\ \mathsf{unit} + \mathsf{var}(\textsc{Var}) + \mathsf{abs}(\textsc{Type},\ X) + \mathsf{app}(X,\ X) + \mathsf{tabs}(X) + \mathsf{tapp}(X,\ \textsc{Type}),$$

where $\textsc{Type}$ is defined in a similar way and $\textsc{Var}$ represents natural-number de Bruijn indices.

This already admits useful 2-way descriptions like

$$\diamond\mathsf{app}(\diamond\mathsf{abs}(\top,\ \top),\ \top) \text{ and } \diamond\mathsf{app}(\diamond\mathsf{app}(\top,\ \top),\ \top),$$

which capture relationships between lambda abstractions and applications. In Section 6.1, we use descriptions like these to find bugs in an evaluator for System F expressions; they ensure that our test suite adequately covers different nestings of abstractions and applications that might provoke bugs.

With a little domain-specific knowledge, we can make the descriptions capture even more. When setting up our case study in Section 6.2, which searches for bugs in GHC's strictness analyzer, we found that it was often useful to track coverage of the *seq* function, which takes two functions as arguments, executes the first for any side-effects (e.g., exceptions), and then executes the second. Modifying our regular expression type to include *seq* as a first-class constructor results in 2-way descriptions now include interactions like

$$\Diamond\mathsf{seq}(\Diamond\mathsf{app}(\top,\ \top),\ \top)$$

that encode interactions of *seq* with other System F constructors. These interactions are crucial for finding bugs in a strictness analyzer, since *seq* gives fine-grained control over the evaluation order within a Haskell expression.

## 5 Thinning Generators with QuickCover

Having generalized the definition of combinatorial coverage to structured data types, the next step is to explore ways of using coverage to improve property-based testing.

When we first approached this problem, we planned to follow the conventional combinatorial testing methodology of generating *covering arrays* [38], i.e., test suites with 100% $t$-way coverage for a given $t$. Rather than use an unbounded stream of random tests—the standard methodology in property-based testing—we would test properties using just the tests in some pre-generated covering array. However, we encountered two major problems with this approach. First, as $t$ grows, covering arrays become frighteningly expensive to generate. While there are efficient methods for generating covering arrays in special cases like 2-way coverage [8], general algorithms for generating compact covering arrays are complex and often slow [23]. Second, we found that covering arrays for sets of test descriptors in the format described above did not do particularly well at finding bugs! In a series of preliminary experiments with one of our case studies, we found that with 4-way coverage (the highest we could generate in reasonable time), our covering arrays did not reliably catch all of the bugs in our test system. Fortunately, after some more head scratching and experimenting, we discovered an alternate approach that works quite well. The trick is to embrace the randomness that makes property-based testing so effective.

In the remainder of this section, we first present an algorithm that uses combinatorial coverage to "thin" a random generator, guiding it to more interesting inputs. Rather than generating a fixed set of tests in the style of covering arrays, this approach produces an unbounded stream of interesting test inputs. Then we discuss some concrete details behind *QuickCover*, the Haskell implementation of our algorithm that we used to obtain the experimental results in Section 6.

### 5.1 Online Generator Thinning

The core of our algorithm is *QuickCheck*'s standard generate-and-test loop. Given a test generator `gen` and a property `p`, *QuickCheck* generates inputs repeatedly until either (1) the property fails, or (2) a time limit is reached.

```
QuickCheck(gen, p):
  repeat LIMIT times:
    # Generate 1 new input
    x = gen()
    # Check the property
    if !p(x), return False
  return True
```

LIMIT is chosen based on the user's specific *testing budget*, and it can vary significantly in practice. In the experiments below, we know *a priori* that a bug exists in the program, so we set LIMIT to infinity and just run tests until the property fails.

Our algorithm modifies this basic one to use combinatorial coverage information when choosing the next test to run.

```
QuickCover(strength, fanout, gen, p):
  coverage = initCoverage()
  repeat LIMIT times:
    # Generate fanout potential inputs
    xs = listOf(gen(), fanout)
    # Find the input with the best improved coverage
    x = argmax[x in xs](
      coverageImprovement(x, coverage, strength) )
    # Check the property
    if !p(x), return False
    # Update the coverage information
    coverage = updateCoverage(x, coverage, strength)
  return True
```

The key idea is that, instead of generating a single input at each iteration, we generate several (controlled by the parameter `fanout`) and select the one that increases combinatorial coverage the most. We test the property on that input and, if it does not fail, update the coverage information based on the test we ran and keep going.

This algorithm is generic with respect to the representation for coverage information, but the particular choice of data structure and interpretation makes a significant difference in both efficiency and effectiveness. In our implementation, coverage information is represented by a multi-set of descriptions:

```
initCoverage():
  return emptyMultiset()

coverageImprovement(x, coverage, strength):
  ds = descriptions(x, strength)
  return sum([ 1 / (count(d, coverage) + 1)
               for d in ds ])

updateCoverage(x, coverage, strength):
  return union(descriptions(x, strength), coverage)
```

At the beginning, the multi-set is empty; as testing progresses, each test is evaluated based on `coverageImprovement`. If a description $d$ had previously been covered $n$ times, it contributes $\frac{1}{n+1}$ to the score. For example, if a test input covers $d_1$ and $d_2$, where previously $d_1$ was not covered and $d_2$ was covered 3 times, the total score for the test input would be $1 + 0.25 = 1.25$.

At first glance, one might think of a simpler approach based on sets instead of multi-sets. Indeed, this was the first thing we tried, but it turned out to perform substantially worse than the multiset-based one in our experiments. The reason is that just covering each description once turns out not to be sufficient to find all bugs, and, once most descriptions have been covered, this approach essentially degenerates to normal random testing. By contrast, the multi-set representation continues to be useful over time; after each description has been covered once, the algorithm begins to favor inputs that cover descriptions a second time, then a third time, and so on. This allows *QuickCover* to generate arbitrarily large test suites that continue to benefit from combinatorial coverage.

Keeping track of coverage information like this does create some overhead.[4] For each test that *QuickCover* considers (including those that are never run), it needs to analyze which descriptions the test covers and check those against the current multi-set. This overhead means that *QuickCover* is often much slower than *QuickCheck* with respect to to generating tests. In the next section, we explore use cases for *QuickCover* that overcome this overhead by running fewer tests.

### 6 Evaluation

Since *QuickCover* adds some overhead to generating tests, one might expect that it will be particularly well suited to situations where each test may be run many times. The primary goal of our experimental evaluation was to test this hypothesis.

[4] The overhead introduced is highly variable and based largely on the exact implementation of the underlying test generator. Appendix A goes into slightly more detail on the asymptotics, but broadly speaking the time it *QuickCover* to generate a test is linear in the fan-out and exponential in the coverage strength.

Of course, running the same test repeatedly *on the same code* is pointless: if it were ever going to fail, it would do so on the first run (ignoring the thorny possibility of "flaky tests" due to nondeterminism [25]). However, running the same test on *successive versions of the code* is not only useful; it is standard practice in two common settings: *regression testing*, i.e., checking that code is still working after changes, and especially *continuous integration*, where regression tests are run automatically every time a developer checks in a new version of the code. In these settings, the overhead introduced by generating many tests and discarding some without running them can be amortized, since the same tests may be reused very many times, so that the cost of generating the test suite becomes less important than the cost of running it.

In order to validate this theory, we designed two experiments using *QuickCover*. The primary goal of these experiments was to answer the question: Does *QuickCover* actually reduce the number of tests needed to find bugs in a real system?

Both case studies answer this question in the affirmative. The first case study, in particular, demonstrates a situation where *QuickCover* needs an average 10× fewer tests to find bugs, compared to pure random testing. We choose an evaluator for System F terms as our example because it allows us to test how *QuickCover* behaves in a small but realistic scenario that requires a fairly complex random testing setup. Our second case study expands on results from Pałka et al. [32], scaling up and applying *QuickCover* to find bugs in the Glasgow Haskell Compiler (GHC) [27].

A secondary goal of our evaluation was to understand whether the generator thinning overhead is *always* too high to make *QuickCover* useful for real-time property-based testing, or if there are any cases where using *QuickCover* would yield a wall-clock improvement even if tests are only run once. Our second case study answers this question in the affirmative.

#### 6.1 Case Study: Normalization Bugs in System F

Our first case study uses combinatorial coverage to thin a highly tuned and optimized test generator for System F [12, 35] terms. The generator produces well-typed System F terms by construction (no mean feat on its own) and is tuned to produce a highly varied distribution of different terms. Despite all the care put into the base generator, we found that modifying the test distribution using *QuickCover* results in a test suite that finds bugs with many fewer inputs.

Generating "interesting" programs (for finding compiler bugs, for example) is an active research area. For instance, a generator for well-typed simply typed lambda-terms has been used to reveal bugs in GHC [6, 20, 32], while a generator for C programs that avoid "undefined behaviors" has been used to find many bugs in production compilers [24, 34, 41] The cited studies are all examples of *differential testing*, where different compilers (or different versions of the same compiler) were run against each other on the same inputs to reveal discrepancies. Similarly, for the present case study we tested different evaluation strategies for System F, comparing the behavior of various buggy versions to a reference implementation.

Recall the definition of System F from Section 4.2. Let $e[v/n]$ stand for substituting $v$ for variable $n$ in $e$, and $e \uparrow_n$ for "lifting"—incrementing the indices of all variables above $n$ in $e$. Then, for example, the standard rule for substituting a type $\tau$ for variable $n$ inside a type abstraction $\Lambda. e$ requires lifting $\tau$ and incrementing the de Bruijn index of the variable being substituted by one:

$$(\Lambda. e)[\tau/n] = \Lambda. e[\tau \uparrow_0 /n + 1]$$

Here are two ways to get this wrong: forget to lift the variables, or forget to increment the index. Those bugs would lead to the following erroneous definitions (the missing operation is shown in red):

$$(\Lambda. e)[\tau/n] = \Lambda. e[\tau \uparrow_0 /n + 1] \quad \text{and} \quad (\Lambda. e)[\tau/n] = \Lambda. e[\tau \uparrow_0 /n + 1].$$

Inspired by errors like these (specifically in the substitution and variable lifting functions), we inserted bugs by hand to create 19 "mutated" versions of two different evaluation relations. (The bugs are described in detail in Appendix C.) The two evaluation relations simplify terms in slightly different ways: the first implements standard big-step evaluation (`eval`), and the second uses a parallel evaluation relation to fully normalize terms (`peval`). (We chose to check both evaluation orders, since some mutations only cause a bug in one implementation or the other.) Since we were interested in bugs in either evaluation order, we tested a joint property:

```
eval e == eval_mutated e && peval e == peval_mutated e
```

Starting with a highly tuned generator for System F terms as our baseline, we used both *QuickCheck* and *QuickCover* to generate a stream of test values for `e` and measured the average number of tests required to find a bug (i.e., Mean-Tests-To-Failure, or MTTF) for each approach.

Surprisingly, we found little or no difference in MTTF between 2-way, 3-way, and 4-way testing, but changing the fan-out did make a large impact. Figure 1 shows both absolute MTTF for various choices of fan-out ($\log_{10}$ scale) and the performance improvement as a ratio of un-thinned MTTF to thinned MTTF. All choices of fan-out produced better MTTF results than the baseline, but higher values of fan-out tended to be more effective on average. In our best experiment, a fan-out of 30 found a bug in an average of 15× fewer tests than the baseline; the overall average was about 10× better. Figure 2 shows the total MTTF improvement across 19 bugs, compared to the maximum theoretical improvement. If our algorithm were able to perfectly pick the best test input every time, the improvement would be proportional to the fan-out (i.e., it is impossible for our algorithm be more than 10× better with a fan-out of 10). On the other hand, if combinatorial coverage were irrelevant to test failure, then we would expect the *QuickCover* test suites to have the *same* MTTF as *QuickCheck*. It is clear from the figure that *QuickCover* is really quite effective in this setting: for small

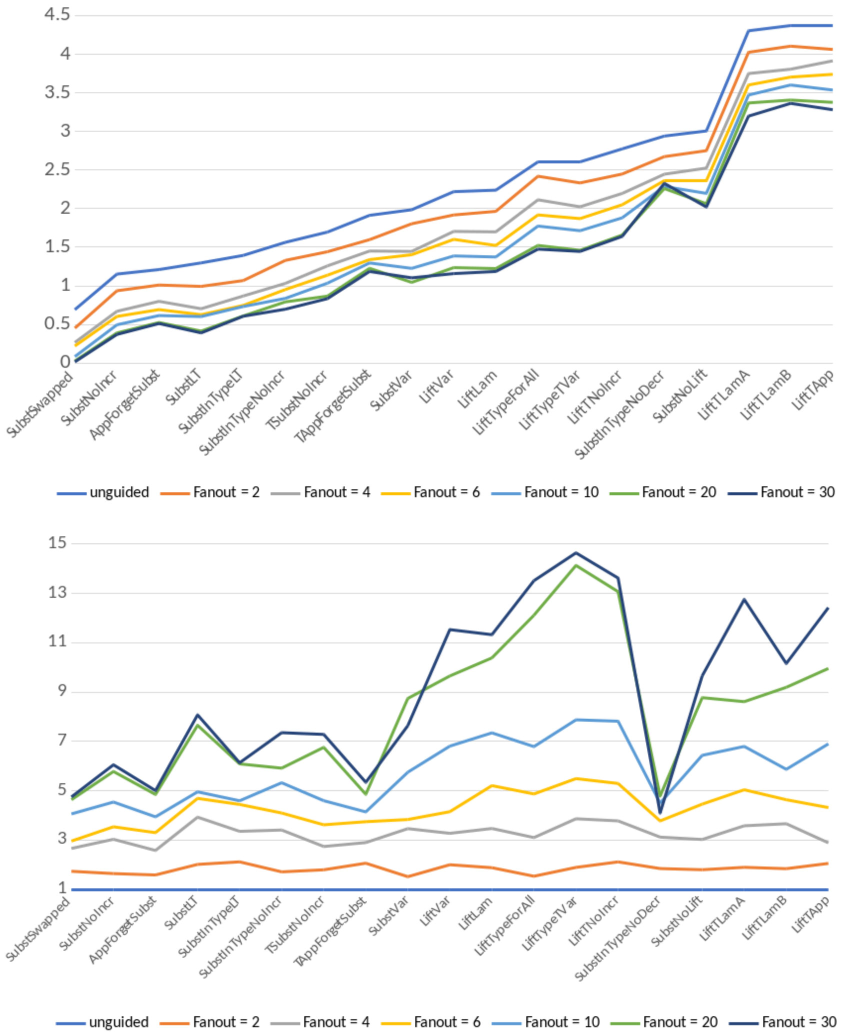

**Fig. 1.** Top: System F MTTF, $\log_{10}$ scale, plotted in order of MTTF for un-thinned random tests, $t = 2$. Bottom: System F MTTF ratio of MTTF for un-thinned random tests to MTTF for *QuickCover*, $t = 2$.

fan-outs, it is very close to the theoretical optimum, and with a fan-out of 30 it achieves about $\frac{1}{3}$ of the potential improvement—that is, three *QuickCover* test cases are more likely to provoke a bug than thirty *QuickCheck* ones.

#### 6.2 Case Study: Strictness Analysis Bugs in GHC

To evaluate how our approach scales, and to investigate whether *QuickCover* can be used not only to reduce the number of tests required but also to speed up bug-finding, we replicated the case study of Pałka et al. [32], which found bugs in the

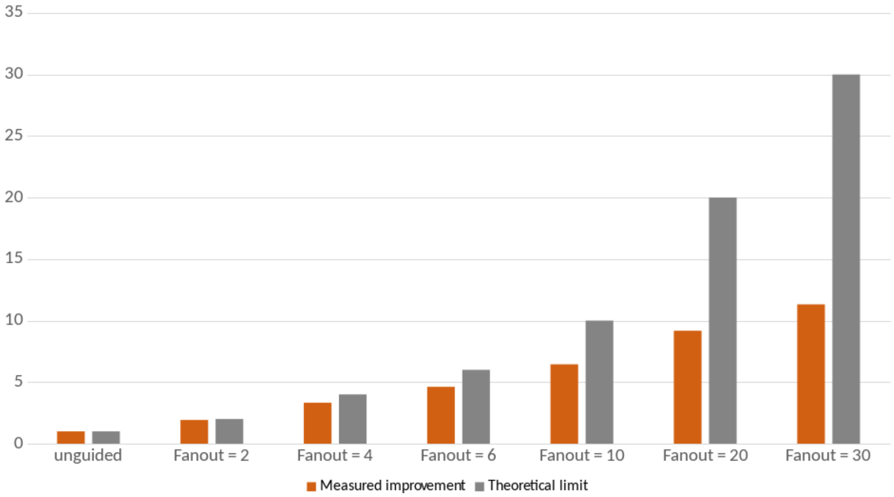

**Fig. 2.** System F, proportional reduction in total number of tests needed to find all bugs.

strictness analyzer of GHC 6.12 using a hand-crafted generator for well-typed lambda terms; we replicated their experimental setup, but used *QuickCover* to thin their generator and produce better tests.

Two attributes of this case study make it an excellent test of the capabilities of our combinatorial thinning approach. First, it found bugs in a real compiler by generating random well-typed lambda terms, and therefore we can evaluate whether the reduction in number of tests observed in the System F case study scales to a production setting. Second, running a test involves invoking the GHC compiler, a heavyweight external process. As a result, reducing the number of tests required to provoke a failure should (and does) lead to an observable improvement in terms of wall-clock performance.

Concretely, Pałka et al. generate a list of functions that manipulate lists of integers and compare the behavior of these functions on partial lists (lists with undefined elements or tails) when compiled with and without optimizations, another example of differential testing. They uncover errors in the strictness analyzer component of GHC's optimizer that lead to inconsistencies where the un-optimized version of the compiled code correctly fails with an error while the optimized version prints something to the screen before failing:

| Input | $-O0$ Output | $-O2$ Output |
|---|---|---|
| `[undefined]` | `Exception` | `[Exception]` |
| `[1,undefined]` | `Exception` | `[1,Exception]` |
| `[1,2,undefined]` | `Exception` | `[1,2,Exception]` |

Finally, to balance the costly compiler invocation with the similarly costly smart generation process, Pałka et al. group 1000 generated functions together in a single module to be compiled; this number was chosen to strike a precise 50-50 balance between generation time and compilation/execution time for each generated module. Since our thinning approach itself introduces approximately a 25% overhead in generation time, we increased the number of tests per module to 1250 to maintain the same balance and make a fair comparison.

We ran our experiments in a Virtual Box running Ubuntu 12.04 (a version old enough to allow for executing GHC 6.12.1), with 4GB RAM in a host machine running i7-8700 @ 3.2GHz. We performed 100 runs of the original case study and 100 runs of our variant that adds combinatorial thinning, using a fan-out of 2 and a strength of 2. We found that our approach reduces the mean number of *tests* required from $21268 \pm 1349$ to $14895 \pm 1056$, a 42% improvement, and reduces the mean *time* to failure from $193 \pm 13$ seconds to $149 \pm 12$, a 30% improvement.

### 7 Related Work

A detailed survey of the (vast) combinatorial testing literature can be found in [30]. Here we discuss just the most closely related work, in particular, other attempts to generalize combinatorial testing to structured and infinite domains. We also discuss other approaches to property based testing with similar goals to to ours, such as adaptive random testing and coverage-guided fuzzing.

#### 7.1 Generalizations of Combinatorial Testing

Salecker and Glesner [37] extend combinatorial testing to sets of terms generated by a context-free grammar. Their approach cleverly maps context-free grammar derivations up to some depth $k$ to sets of parameter choices; then it uses standard full-coverage test suite generation algorithms to pick a subset of derivations to test. The main limitation of this approach is the parameter $k$. By limiting the derivation depth, this approach only defines coverage over a finite subset of the input type. By contrast, our definition of coverage works over infinite types by exploiting the recursive nature of the $\diamond$ operator. We focus on description size rather than term size, which provides more flexibility for "packing" multiple descriptions into a single test.

Another approach to combinatorial testing of context-free inputs is due to Lämmel and Schulte [19]. Their system also uses a depth bound, but it provides the user finer-grained control. At each node in the grammar, the user is free to limit the coverage requirements and prune unnecessary tests. This is an elegant solution for situations where the desired interactions are known *a priori*. Unfortunately, this approach needs to be re-tuned manually to every specific type and use-case, so it is not the general solution we were after.

Finally, Kuhn et al. [15] present a notion of *sequence covering arrays* to describe combinatorial coverage of sequences of events. We believe that $t$-way sequence covering arrays in their system are equivalent to $(2t-1)$-way full-coverage test suites of the appropriate list type in ours. They also have a reasonably efficient algorithm for generating covering arrays in this specialized case.

Our idea to use regular tree expressions for coverage is partly inspired by Usaola et al. [40] and Mariani et al. [26]. Rather than generate a set of terms to cover an ADT, these works generate strings to cover (i.e. match in every possible way) a particular regular expression. This turns out to be quite a different problem, but these explorations led us to consider coverage in context of of formal languages.

#### 7.2 Comparison with Enumerative Property-Based Testing

Another approach to property-based testing research is based on enumeration of small test cases, rather than random generation. Tools like *SmallCheck* [36] offer guarantees that there is no counterexample smaller than a certain limit, and moreover always report the smallest counterexample when it exists. To compare our approach with this type of tool, we repeated our System F evaluation with a variety of enumerative testing tools.

We first tried SmallCheck, which enumerates all test cases up to a given *depth*. Unfortunately, the number of System F terms rises very rapidly with the depth: SmallCheck quickly enumerated 708 terms of depth up to three, but could not enumerate all terms of depth four within 20 minutes of CPU time.[5] Only one of the 19 bugs we planted was provoked by any of those 708 terms.

However, SmallCheck wastes effort generating syntactically correct terms that are not type correct; only 140 of the 708 were well-typed. *Lazy SmallCheck* [36] exploits laziness in property preconditions to discard many test cases in a group—in this case, all those terms that fail a type-check in the same way are discarded together. Because well-typedness is such a strong precondition, Lazy SmallCheck is able to dramatically reduce the number of terms needed at each depth, enabling us to increase the depth limit to 4, and generate over five million terms. The result was a much more comprehensive test suite than normal SmallCheck, but it still only found 8 out of our 19 bugs.

The problem here is that the smallest counterexamples we are searching for are quite small terms, but may nevertheless have a few fairly deep nodes in their syntax trees. More recent enumerative tools, such as LeanCheck [3], enumerate test cases in *size* order, instead of in *depth* order, thus reaching terms with just a few deeper nodes much earlier in the enumeration. For this example, LeanCheck runs out of memory after about 11 million tests. but this was enough to find all but four of the planted bugs.

However, LeanCheck does not use the Lazy SmallCheck optimization, and so is mostly testing ill-typed terms, for which our property holds vacuously. *SciFe* [18] enumerates in size order *and* uses the Lazy SmallCheck optimization, with good results. It is hard to apply SciFe, which is designed to test Scala, to our Haskell code, so instead we created a Lazy SmallCheck variant that enumerates in size order. With this variant, we could find all of the planted bugs, with counterexample sizes varying from 5 to 14. Lazy SmallCheck does not report the number of tests needed to find a counterexample, just the size at which it was found, together with the number of test cases of each size. We can therefore only

[5] Compiled with ghc -O2, on an Intel i7-6700k with 32GB of RAM under Windows 10.

give a lower bound for the number of tests needed to find each bug. Figure 3 plots this lower bound against the average number of tests needed by QuickCheck and by QuickCover. For these bugs, it is clear that the enumerative approach is not competitive with QuickCheck, let alone with QuickCover. The improvement in the numbers of tests needed ranges from 1.7 to 5.5 *orders of magnitude*, with a mean across all the bugs of 3.3 orders of magnitude.

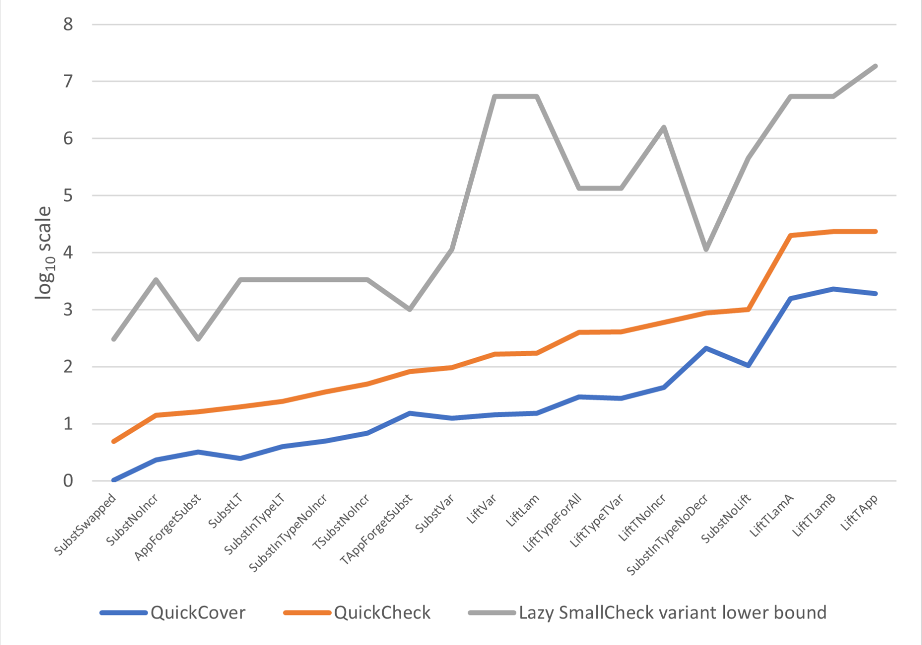

**Fig. 3.** System F MTTF for QuickCheck and QuickCover, and the *lower bound* on the number of tests run by our Lazy SmallCheck variant, $\log_{10}$ scale.

#### 7.3 Comparison with Fuzzing Techniques

Coverage-guided fuzzing tools like AFL [22] can be viewed as a way of using a different form of feedback (branch instead of combinatorial coverage) to improve the generation of random inputs by finding more "interesting" tests. Fuzzing is a huge topic [43] that has exploded in popularity recently, with researchers evaluating the benefits of using more forms of feedback [13, 31], incorporating learning [28,33] or symbolic [39,42] techniques, and bringing the benefits of these methods to functional programming [11, 21]. One fundamental difference, however, is that all of these techniques are *online* and *grey-box*: they instrument and execute the program on various inputs in order to obtain feedback. In contrast, combinatorial coverage can be computed without any knowledge of the code itself, therefore providing a convenient black-box alternative that can be valuable when the same test suite is to be used for many versions of the code (such as in regression testing) or when executing the code is costly (such as when testing production compilers).

Chen et al.'s *adaptive random testing* (ART) [4] uses an algorithm that, like *QuickCover*'s, generates a set of random tests and selects the most interesting to run. Rather than using combinatorial coverage, ART requires a *distance metric* on test cases—at each step, the candidate which is farthest from the already-run tests is selected. Chen et al. show that this approach finds bugs after fewer tests, on average, in the programs they study. ART was first proposed for programs with numerical inputs, but Ciupa et al. [5] showed how to define a suitable metric on objects in an object-oriented language and used it to obtain a reduction of up to two orders of magnitude in the number of tests needed to find a bug. Like combinatorial testing, ART is a black-box approach that depends only on the test cases themselves, not on the code under test.

However, Arcuri and Briand [1] question ART's value in practice, because of the quadratic number of distance computations it requires, from each new test to every previously executed test; in a large empirical study, they found that the cost of these computations made ART uncompetitive with ordinary random testing. While our approach also has significant computational overhead, the time and space complexity grow with the number of possible descriptions (derived from the data type definition and the choice of strength) and *not* with the total number of tests run—i.e., testing will not slow down over time. In addition, our approach works in situations where a distance metric between inputs does not make sense.

### 8 Conclusion and Future Work

We have presented a generalized definition of combinatorial coverage and an effective way to use that definition for property-based testing, generalizing the definition of combinatorial coverage to work in the realm of algebraic data types with the help of regular tree expressions. Our sparse test descriptions provide a robust way to look at combinatorial testing, which specializes to the classical approach. We use these sparse descriptions as a basis for *QuickCover*—a tool that thins a random generator to increase combinatorial coverage. Two case studies show that *QuickCover* is useful in practice, finding bugs using an average of 10× fewer tests.

The rest of this section sketches a number of potential directions for further research.

#### 8.1 Variations

Our experiments show that sparse test descriptions are a good way to define combinatorial coverage for algebraic data types, but they are certainly not the the only way. Here we discuss some variations and why they might be interesting to explore.

*Representative Samples of Large Types* Perhaps it is possible to do combinatorial testing with ADTs by having humans decide exactly which trees to cover. This approach is already widely used in combinatorial testing to deal with types like machine integers that, though technically finite, are much too large for testing to efficiently cover all their "constructors." For example, if a human tester knows (by reading the code, or because they wrote it) that it contains an if-statement guarded by `x < 5`, they might choose to cover

$$\mathtt{x} \in \{-2147483648,\ 0,\ 4,\ 5,\ 6,\ 2147483647\}.$$

The tester might choose values around 5 because those are important to the specific use case and boundary values and 0 to check for common edge-cases. Concretely, this practice means that instead of trying to cover $\mathsf{tuple}_3(\textsc{Int},\ \mathsf{true}+\mathsf{false},\ \mathsf{true}+\mathsf{false})$, the tester covers the specification

$$\mathsf{tuple}_3(-2147483648 + 0 + 4 + 5 + 6 + 2147483647,\ \mathsf{true}+\mathsf{false},\ \mathsf{true}+\mathsf{false}).$$

In our setting, this might mean choosing a representative set of *constructor trees* to cover, and then treating them like a finite set. In much the same way as with integers, rather than cover

$$\mathsf{tuple}_3(\tau_{\mathsf{list(bool)}},\ \mathsf{true}+\mathsf{false},\ \mathsf{true}+\mathsf{false}),$$

we could treat a selection of lists as atomic constructors, and cover the specification

$$\mathsf{tuple}_3(\boxed{[]} + \boxed{[\mathsf{true},\ \mathsf{false}]} + \boxed{[\mathsf{false},\ \mathsf{false},\ \mathsf{false}]},\ \mathsf{true}+\mathsf{false},\ \mathsf{true}+\mathsf{false})$$

which has 2-way descriptions like

$$\mathsf{tuple}_3(\boxed{[]},\ \top,\ \mathsf{false}) \quad \text{and} \quad \mathsf{tuple}_3(\boxed{[\mathsf{true},\ \mathsf{false}]},\ \mathsf{true},\ \top).$$

Just as testers choose representative sets of integers, they could choose sets of trees that they think are interesting and only cover those trees. Of course, the set of all trees for a type is usually much larger and more complex than the set of integers, so this approach may not be as practical for structured types as for integers. Still, it is possible that small amounts of human intervention could help guide the choice of descriptions to cover.

*Type-Tagged Constructors* Another variation to our approach would change the way that ADTs are translated into constructor trees. In Appendix B we show a simple example of a `Translation` for lists of Booleans, but an interesting problem arises if we consider *lists of* lists of Booleans. The most basic approach would be to use the same constructors (`LCNil` and `LCCons`) for both "levels" of list. For example, `[[True]]` would become (with a small abuse of notation)

```
LCCons (LCCons LCTrue LCNil) LCNil.
```

Depending on the application, it might actually make more sense to use different constructors for the different list types (`[Bool]` vs. `[[Bool]]`). For example, `[[True]]` could instead be translated as

```
LCOuterCons (LCInnerCons LCTrue LCInnerNil) LCInnerNil
```

(with a slight abuse of notation), allowing for a broader range of potential test descriptions. This observation can be generalized to any polymorphic ADT: any time a single constructor is used at multiple types, it is likely beneficial to differentiate between them by translating to constructor tree nodes tagged with a monomorphized type.

*Pattern Descriptions* A third potential variation is a modification to make test descriptions a bit less sparse. Recall that sparse test descriptions are defined as

$$d \triangleq \top \mid \diamond C(d_1, \ \ldots, \ d_n).$$

What if we chose this instead?

$$d \triangleq \diamond d'$$
$$d' \triangleq C(d'_1, \ \ldots, \ d'_n)$$

In the former case, every relationship is "eventual": there is never a requirement that a particular constructor appear *directly* beneath another. In the latter case, the descriptions enforce a direct parent-child relationship, and we simply allow the expression to match anywhere in the term. We might call this class "pattern" test descriptions.

We chose sparse descriptions for this work because putting $\diamond$ before every constructor leaves more opportunities for nodes matching different descriptions to be "interleaved" within a term, leading to smaller test suites in general. In some small experiments, this alternative proposal seemed to perform similarly across the board but worse in a few cases. Even so, experimenting with the use of eventually in descriptions might lead to interesting new ideas.

#### 8.2 Combinatorial Coverage of More Types

Our sparse tree description definition of combinatorial coverage is focused on inductive algebraic types. While these encompass a wide range of the types that functional programmers use, it is far from everything. One interesting extension would generalize descriptions to co-inductive types. We actually think that the current definition might almost suffice—regular tree expressions can denote infinite structures, so this generalization would likely only affect our algorithms and the implementation of *QuickCover*. We also should be able to include Generalized Algebraic Data Types (GADTs) without too much hassle. The biggest unknown is function types, which seem to require something more powerful than regular tree expressions to describe; indeed, it is not clear that combinatorial testing even makes sense for higher-order values.

#### 8.3 Regular Tree Expressions for Directed Generation

As we have shown, regular tree expressions are a powerful language for picking out subsets of types. In this paper, we mostly focused on automatically generating small descriptions, but it might be possible to apply this idea more broadly for specifying sets of tests. One straightforward extension would be to use the same machinery that we use for *QuickCover* but, instead of covering an automatically generated set of descriptions, ensure that, at a minimum, some manually specified set of expressions is covered. For example, we could use a modified version of our algorithm to generate a test set where

$$\mathsf{nil},\ \mathsf{cons}(\top,\ \mathsf{nil}),\ \text{and}\ \mu X.\ \mathsf{cons}(\mathsf{true},\ X) + \mathsf{nil}$$

are all covered. (Concretely, this would be a test suite containing, at a minimum, the empty list, a singleton list, and a list containing only true.) This might be useful for cases where the testers know *a priori* that certain shapes of inputs are important to test, but they still want to explore random inputs with those shapes.

A different approach would be to create a tool that synthesizes *QuickCheck* generators that only generate terms matching a particular regular tree expression. This idea, related to work on adapting *branching processes* to control test distributions [29], would make it easy to write highly customized generators and meticulously control the generated test suites.

**Acknowledgments**

Thank you to Calvin Beck, Filip Niksic, and Irene Yoon for help with the early development of these ideas. Thanks to Kostis Sagonas, Alexandra Silva, and Andrew Hirsch for feedback along the way. This work was supported by NSF awards #1421243, *Random Testing for Language Design* and #1521523, *Expeditions in Computing: The Science of Deep Specification*, by the Defense Advanced Research Projects Agency (DARPA) under Contract No. HR0011-18-C-0011, by the United States Air Force and DARPA under Contract No FA8750-16-C-0022 (any opinions, findings and conclusions or recommendations expressed in this material are those of the authors and do not necessarily reflect the views of Vetenskapsrådet, the NSF, the United States Air Force, or DARPA), and by Vetenskapsrådet in Sweden for funding under the SyTeC project (grant number 2016-06204).
